# Emodin Induced SREBP1-Dependent and SREBP1-Independent Apoptosis in Hepatocellular Carcinoma Cells

**DOI:** 10.3389/fphar.2019.00709

**Published:** 2019-06-25

**Authors:** Nian Yang, Chen Li, Hongliang Li, Ming Liu, Xiaojun Cai, Fengjun Cao, Yibin Feng, Minglun Li, Xuanbin Wang

**Affiliations:** ^1^Laboratory of Chinese Herbal Pharmacology, Oncology Center, Renmin Hospital, Hubei University of Medicine, Shiyan, China; ^2^Hubei Key Laboratory of Wudang Local Chinese Medicine Research, Biomedical Research Institute, Hubei University of Medicine, Shiyan, China; ^3^Department of Pharmacy, Jurong Hospital Affiliated to Jiangsu University, Zhenjiang, China; ^4^School of Chinese Medicine, The University of Hong Kong, Hong Kong, China; ^5^Department of Radiation Oncology, University Hospital, LMU, Munich, Germany

**Keywords:** emodin, lipid metabolism, SREBP1, intrinsic apoptosis, hepatocellular carcinoma cells

## Abstract

*Reynoutria multiflora* (Thunb.) Moldenke (He Shou Wu) has been used for about 20 centuries as a Chinese medicinal herb for its activities of anticancer, anti-hyperlipidemia, and anti-aging. Previously, we found that He Shou Wu ethanol extract could induce apoptosis in hepatocellular carcinoma cells, and we also screened its active components. In this study, we investigated whether lowering lipid metabolism of emodin, a main active component in He Shou Wu, was associated with inhibitory effects in hepatocellular carcinoma cells. The correlation of apoptosis induction and lipid metabolism was investigated. The intrinsic apoptotic cell death, lipid production, and their signaling pathways were investigated in emodin-treated human hepatocellular carcinoma cells Bel-7402. The data showed that emodin triggered apoptosis in Bel-7402 cells. The mitochondrial membrane potential (ΔΨm) was reduced in emodin-treated Bel-7402 cells. We also found that emodin activated the expression of intrinsic apoptosis signaling pathway-related proteins, cleaved-caspase 9 and 3, Apaf 1, cytochrome c (CYTC), apoptosis-inducing factor, endonuclease G, Bax, and Bcl-2. Furthermore, the level of triglycerides and desaturation of fatty acids was reduced in Bel-7402 cells when exposed to emodin. Furthermore, the expression level of messenger RNA (mRNA) and protein of sterol regulatory element binding protein 1 (SREBP1) as well as its downstream signaling pathway and the synthesis and the desaturation of fatty acid metabolism-associated proteins (adenosine triphosphate citrate lyase, acetyl-CoA carboxylase alpha, fatty acid synthase (FASN), and stearoyl-CoA desaturase D) were also decreased. Notably, knock-out of *SREBP1* in Bel-7402 cells was also found to induce less intrinsic apoptosis than did emodin. In conclusion, these results indicated that emodin could induce apoptosis in an SREBP1-dependent and SREBP1-independent manner in hepatocellular carcinoma cells.

## Introduction

*Reynoutria multiflora* (Thunb.) Moldenke, a type of Chinese medicine and a Taoist medicine, was named as Maganshi (马肝石) in the era of Eastern Han Dynasty (25–220 AD) and after a long-lived man in Tang Dynasty (618–907 AD), He Shou Wu (何首乌), in the legend of Chinese Medical Work, Compendium of Materia Medica (本草纲目) (Li, 2016). In Chinese folk medicine philosophy, the root of He Shou Wu tonifies the liver and kidney, boosts essence blood, blackens the beard and hair, strengthens sinew and bone, transforms turbidity, and reduces lipid levels, which acts to protect the liver, bone, sexual and reproductive functions, improve memory and intelligence, and promote antiaging, lipid lowering, and anticancer qualities (Chen, 2017). Taoists preferred it because of its antiaging effects (Shang, 2004). He Shou Wu consisted of 2,3,5,4’-tetrahydroxystilbene-2-O-β-D-glucoside, anthraquinones (Lin et al., 2015; Li H. et al., 2016) and other active compounds. We previously found that the ethanol extract of processed He Shou Wu (HSWE) induces apoptosis and inhibits lipogenesis in human hepatocellular carcinoma (HCC) cells by inhibiting sterol regulatory element binding protein 1 (SREBP1).

A growing body of evidence suggested that many human cancers emerge as alterations in lipid metabolism and *de novo* lipogenesis was essential for tumor growth, survival, and resistance to therapies. Increased SREBP-1 and lipogenic enzymes transcriptionally activated by SREBP1 have been found in tumor patients (Huang et al., 2012; Pandey et al., 2013; Li et al., 2014). SREBP1 regulates the expression of genes associated with fatty acid synthesis (Edwards et al., 2000; Moon et al., 2001). When intracellular unsaturated fatty acids or sterols are depleted, concomitant cleavage in the Golgi bodies by two site-specific proteases occurs, and the mature form of the N-terminal protein (mSREBP1) is released and enters the nucleus to activate transcription of target genes such as ACLY, ACACA, FASN, and SCD (Zhao et al., 2014) with sterol regulatory element sequences in their promoters (Horton, 2002). In the pathway of fatty acid metabolism, ACLY, ACACA, and FASN are the key enzymes in the synthesis of fatty acids. ACLY converts mitochondrial citric acid to oxaloacetate and acetyl-CoA, the precursor for fatty acid synthesis. Next, ACACA carboxylates acetyl-CoA to form malonyl-CoA, a substrate for fatty acid synthesis. In turn, FASN catalyzes successive condensation polymerizations to form a fatty acid from malonyl-CoA and acetyl-CoA substrates, generating mainly long-chain fatty acid palmitic acid (Currie et al., 2013). It has been reported that specific blocking of the FASN expression led to an accumulation of malonyl-CoA, resulting in apoptosis induction (Bandyopadhyay et al., 2006). Regarding fatty acid desaturation, SCD is a subtype of the Δ9 fatty acid desaturation-limiting enzyme family that can catalyze saturated fatty acids (SFAs, including palmitic acid and stearic acid) to form monounsaturated fatty acids (MUFAs, including palmitoleic acid and oleic acid) (Mele et al., 2007; Angelucci et al., 2015). SFAs and MUFAs are the basic elements of membrane phospholipids (Evans et al., 2009). Once the expression of SCD is inhibited, it can result in the imbalance between mitochondrial SFAs and MUFAs, leading to apoptosis (Lewis et al., 2015). Therefore, SREBP1-targeted therapy is expected to be an effective strategy for the treatment of metabolic syndrome and cancer (Guo et al., 2014; Soyal et al., 2015). HSWE inhibited mRNA and protein expression of stearoyl-CoA desaturase (SCD) by blocking its upstream factor SREBP1, inducing a decrease of ratio of SFA and MUFAs, which led to an increased level of reactive oxygen species (ROS), alanine aminotransferase (ALT), and aspartate aminotransferase (AST)and apoptosis, finally (Li J. et al., 2016; Li et al., 2018). We then screened the bioactive components in HSWE and found that emodin (1,3,8-trihydroxy-6-methyl-anthraquinone), a natural anthraquinone derivative, was the major bioactive component (Yang et al., 2018). Studies showed that emodin possessed numerous functions that included arresting cell cycle by blocking cyclin D, cyclin E (Wang et al., 2015), and CDK2 (Zhang et al., 2015); inducing apoptosis by upregulating extrinsic apoptotic fas ligand (FASL) signaling (Li et al., 2014); activating intrinsic apoptotic factors, Cyto c, Caspase-9, and apoptosis-inducing factor (AIF) (Ma et al., 2012); declining the mitochondrial membrane potential (ΔΨm) (Liu et al., 2012); inhibiting migration and invasion by downregulating transforming growth factor-β (TGF-β) (Thacker and Karunagaran, 2015) and Wnt/beta-Catenin signaling pathway (Gu et al., 2019); reversing multidrug resistance by downregulating multidrug resistance-associated protein 1 (MRP1) (Ma et al., 2014), and restricting energy metabolism by inhibiting fatty acid synthase (Lee et al., 2017) in cancers. Moreover, emodin induces both extrinsic and intrinsic apoptosis (Cui et al., 2016). However, it remains unclear whether a decrease in lipids is associated with cancer treatment. However, the underlying mechanism of lipid metabolism regulation and apoptosis induction *via* emodin in HCC cells remains unknown.

In this study, we concentrated on the capacities of emodin on restricting lipid metabolism and inducing apoptosis in HCC cells to broaden insight in the mechanisms of He Shou Wu in preventing and treating cancer.

## Materials and Methods

### Chemicals and Reagents

Emodin (purity: 95.0%) was purchased from Topscience (Shanghai, China). RPMI-1640 was used as the culture medium and was purchased from Gibco (Gaithersburg, MD, USA). Neonatal bovine serum (NBS) was obtained from Tianhang (Hangzhou, China). PE Annexin V-7AAD apoptosis detection kit was supplied by BD Biosciences (San Jose, CA, USA). JC-10 mitochondrial membrane potential kit was purchased from Solarbio (Beijing, China). Triglyceride (TG) Assay Kit was supplied by Jiancheng Bioengineering Institute (Nanjing, China). Oleic acid, stearic acid, palmitoleic acid, and palmitic acid were obtained from Sigma-Aldrich (St. Louis, MO, USA). Primary antibodies glyceraldehyde 3-phosphate dehydrogenase (GAPDH, 60004-1-Ig, mouse monoclonal, 1:10,000), adenosine triphosphate citrate lyase (ACLY, 15421-1-AP, rabbit polycolonal, 1:1,000), acetyl-CoA carboxylase alpha (ACACA, 21923-1-AP, rabbit polycolonal, 1:500), fatty acid synthase (FASN, 10624-2-AP, rabbit polycolonal, 1:500), SCD (23393-1-AP, rabbit polycolonal, 1:500), B-cell lymphoma-2 (Bcl-2, 60178-1-Ig, mouse monoclonal, 1:1,000), Bcl-2-associated X (Bax, 50599-2-Ig, rabbit polycolonal, 1:2,000), AIF (17984-1-AP, rabbit polycolonal, 1:4,000), endonuclease G (ENDOG) (22148-1-AP, rabbit polycolonal, 1:1,000), Cytochrome c (CYTC, 66264-1-Ig, mouse monoclonal, 1:5,000), and APAF1 (21710-1-AP, rabbit polycolonal, 1:500) were purchased from Proteintech Group (Wuhan, China). Primary antibodies SREBP1 (NB600-582, mouse monoclonal, 1:1,000) were obtained from Novus (Littleton, CO, USA). Primary antibodies for caspase-3 (9662, rabbit polycolonal, 1:1,000), cleaved-caspase-3 (9661, rabbit polycolonal, 1:1,000), caspase-9 (9502, rabbit polycolonal, 1:1,000) and cleaved-caspase-9 (9501, rabbit polycolonal, 1:1,000) as well as secondary antibodies were supplied by Cell Signaling Technology (Danvers, MA, USA).

### Establishment of SREBP1 Overexpression and Knockout HCC Cell Lines

**Figure 1:**
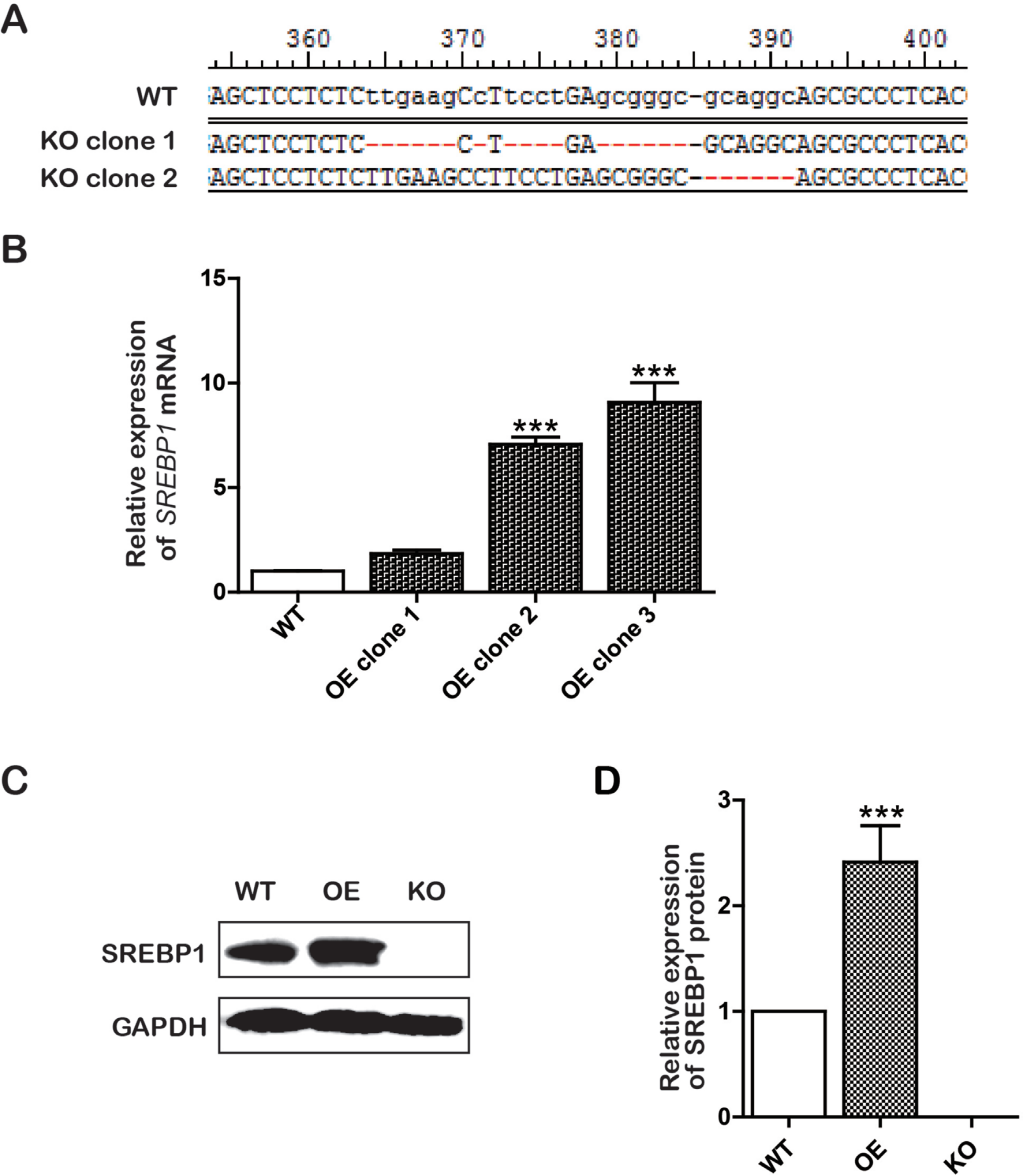
Construction of knockout (KO) and overexpression (OE) cell lines. **(A)** The sequencing results of KO positive clone cells; **(B)** The messenger RNA (mRNA) level of *SREBP1* in wild type (WT) and OE Bel-7402 cells. Data were expressed as mean ± standard error of the mean. ***P < 0.001 vs. WT group; **(C** and **D)** The protein level of SREBP1 in WT, OE, and KO Bel-7402 cells. Data were expressed as mean ± standard error of the mean. ***P < 0.001 vs. WT group.

To construct lentiCRISPRv2/SREBP1-knockout (KO) recombinant plasmid, the sgRNAs targeting SREBP1 were designed according to CRISPR DESIGN (http://crispr.mit.edu/), and the CRISPR KO plasmids were constructed according to ZhangLab’ instructions (Sanjana et al., 2014; Shalem et al., 2014). To generate the SREBP1 overexpression (OE) vector, a fragment containing the complete SREBP1 open reading frame was released from the recombinant plasmid pHAGE-CMV-MCS-IZsGreen/SREBP1 OE by digestion with restriction enzymes *Xho*I and *Bam*HI. Next, the two recombinant plasmids were confirmed by enzyme analysis and DNA sequencing. To obtain the single cell of SREBP1 KO and SREBP1 OE clones, Bel-7402 cells were respectively transfected with two recombinant plasmid according to Lipofectamine™ 3000 (Invitrogen, Carlsbad, CA, USA) instructions. Stable transfectants of SREBP1 KO were screened using puromycin (5.5 μg/mL) for a 3- to 4-day period of selection, and SREBP1 OE was screened by green fluorescence of recombinant plasmid. Next, the two stable transfected cell lines were confirmed by polymerase chain reaction (PCR) and Western blotting analysis. T7 endonuclease 1 (T7E1) assayed SREBP1 KO cell lines additionally (**Figure 1**).

### Cell Culture and Drug Treatment

The HCC cells Bel-7402 (stored in our laboratory) were cultured in RPMI-1640 supplemented with 10% NBS in a humidified incubator (5% CO_2_ and 37°C). The stock solutions of emodin (100 mmol/L) dissolved in dimethyl sulfoxide (DMSO) were diluted to different concentrations as needed.

### MTT Assay

To assess the inhibitory effects of emodin on Bel-7402 cells, cell viability was evaluated by MTT (3-(4,5-dimethylthiazol-2-yl)-2,5 diphenyltetrazolium bromide) assay. Briefly, exponentially growing cells (7 × 10^3^ cells per well) were seeded 24 h prior to treatment into 96-well plates. After treatment with emodin (0, 25, 50, 100, 200, 400, and 600 μmol/L) for 12, 24, and 48 h, cell proliferation was performed by adding 10 μL of MTT solution (5 mg/mL) to each well, and plates were then incubated for 4 h. After dissolving the purple formazan crystals, 150 μL DMSO was added to each well at 37°C for 5 min. The absorbance values at 490 nm were read using a Microplate Reader (BioTek, Winooski, VT, USA).

### PE Annexin V-7AAD Assay

To investigate the apoptosis in emodin-treated Bel-7402 cells, PE Annexin V-7AAD double staining was used, and the apoptosis was assessed using flow cytometry. Briefly, cells (1 × 10^6^ cells per well) were seeded into six-well plates, incubated at 37°C overnight. After treatments with emodin for 24 h, the cells were harvested and washed twice with cold phosphate buffered saline (PBS). Then, the cells were resuspended in Annexin-V binding buffer and stained with PE Annexin V and 7-AAD according to the manufacturer’s protocol. The fluorescence intensity was examined using a flow cytometer (Beckman, Brea, CA, USA). Onset of early and late apoptosis was determined using Annexin-V, whereas 7AAD was used for testing necrosis and late apoptosis.

### JC-10 Assay

JC-10 is a fluorescent probe, and it exists as a red fluorescent dimer under a high mitochondrial potential (ΔΨm). However, under a low ΔΨm, e.g., a condition of mitochondrial injury, it converts into monomeric form and stains cells green. Thus a ratio of red/green JC-10 fluorescence can be used to indicate the change of ΔΨm. A high ratio of red/green JC-10 fluorescence indicates a higher ΔΨm, whereas a low ratio of red/green JC-10 fluorescence means a lower ΔΨm. To analyze the mitochondrial injury by emodin, the ΔΨm was detected using JC-10 assay. Briefly, cells were cultured in 6-well plates at a density of 4 × 10^5^/well and incubated at 37°C overnight. After treatments with emodin for 24 h, the culture medium was removed and the cells were washed twice with PBS. Then, cells were loaded with 1 mL JC-10 water solution (0.5%, v/v) according to the manufacturer’s protocol. JC-10 fluorescence was measured *via* fluorescent microscopy (Olympus, Tokyo, Japan).

### TG Assay

To analyze the alterations of fatty acids contents by emodin, the level of TG was measured. Briefly, cells (1 × 10^6^ cells per well) were seeded 24 h prior to treatment into 6-well plates. After treatments with emodin for 24 h, the cells were collected and washed twice with PBS. Then, resuspended cells were lysed using 100 μL Triton X-100 (2%). The samples were measured according to the manufacturer’s protocol. Results were normalized to total protein content as determined by the bicinchoninic acid (BCA) assay.

### Fatty Acids Assay

To evaluate the lipid metabolism changes, the production ratio of oleic acid/stearic acid and palmitoleic acid/palmitic acid was measured using ultra-performance liquid chromatography-mass spectrometry (UPLC-MS) assay (Waters Corporation, Milford, MA, USA). Briefly, cells (1 × 10^6^ cells per well) were seeded 24 h prior to treatment into six-well plates. After treatment with emodin for 24 h, the cells were harvested and washed twice with PBS. Then, fatty acids were extracted and measured using UPLC-MS assay as mentioned in the previous report (Li et al., 2018). Results were normalized to total protein content as determined using the BCA assay.

### Real-Time Quantitative Polymerase Chain Reaction

To observe the inhibitory effects of emodin on the lipid metabolism-associated genes, the mRNA expressions were detected by the real-time quantitative polymerase chain reaction (RT-qPCR) assay. Total RNA from the cells was isolated using Trizol Reagent (Invitrogen, Carlsbad, CA, USA) and converted into complementary DNA (cDNA) *via* reverse transcription using the ReverTra Ace qPCR RT Kit (TOYOBO, Osaka, Japan) as instructed in the supplier’s protocol. Then, RT-qPCR analysis was performed using SYBR™ Green qPCR supermix-UDG with ROX in a Quantitative PCR System (Applied Biosystems, Foster, CA, USA). Primers used in the RT-qPCR assay are listed in **Table 1**. The following cycling conditions were used: 10 min at 95°C, and 40 cycles of 15 s at 95°C and 1 min at 60°C. Relative expression was statistically evaluated by the 2^−ΔΔCt^ method with normalization against β-actin.

**Table 1:** List of primers used in the study.

Gene	Primer sequence (5’→3’)	Product (bp)
*SREBP1*	Forward 5’-ACACAGCAACCAGAAACTCAAG-3’	153
	Reverse 5’-AGTGTGTCCTCCACCTCAGTCT-3’	
*ACLY*	Forward 5’-GACCTATGACTATGCCAAGACTAT-3’	88
	Reverse 5’-GATGCTGCCTCCAATGATGA-3’	
*ACACA*	Forward 5’-AATAGCGTCTCTAACTTCCTTCAC-3’	200
	Reverse 5’-CCGTCACTCAGCCGATGTA-3’	
*FASN*	Forward 5’-GGACATGGCTTAGAAGTGGAA-3’	164
	Reverse 5’-TTGGTGTTGCTGGTGAGTG-3’	
*SCD*	Forward 5’-GCGATATGCTGTGGTGCTTA-3’	153
	Reverse 5’-GAGTGGTGGTAGTTGTGGAAG-3’	
*β-actin*	Forward 5’-TCGTGCGTGACATTAAGGAG-3’	176
	Reverse 5’-GAAGGAAGGCTGGAAGAGTG-3’	

SREBP1, sterol regulatory element binding protein 1; ACACA, acetyl-CoA carboxylase alpha; ACLY, ATP citrate lyase; FASN, fatty acid synthase; SCD, stearoyl-coenzyme A desaturase.

### Western Blotting Assay

To assess the effects of lipid metabolism and apoptosis-associated proteins by emodin, the expression of proteins was detected by Western blotting. After planned treatments, cells were collected and lysed in radio immunoprecipitation assay (RIPA) buffer [1% Triton X-100, 1% deoxycholate, 0.1% sodium dodecyl sulfate (SDS)]. Equal amounts of total proteins were separated by appropriate SDS-polyacrylamide gel electrophoresis (SDS-PAGE) followed by transferring to polyvinylidene difluoride (PVDF) membranes. After blocking with 5% bovine serum albumin, the membrane was incubated with specific primary antibodies and the corresponding second antibodies, respectively. The specific protein bands were visualized with Millipore enhanced chemiluminescence kit and imaged using ChemiDocTM XRS+ Molecular Imager (Bio-Rad, Hercules, CA, USA).

### Statistical Analysis

All experiments were performed separately at least three times. The significance of intergroup differences was evaluated by one-way analysis of variance using the GraphPad Prism 5.0 software (GraphPad Software, La Jolla, CA, USA). Values of *P* < 0.05 were considered as significant.

## Results

### Emodin Inhibited Cell Proliferation and Induced Intrinsic Apoptosis in Bel-7402 Cells

**Figure 2:**
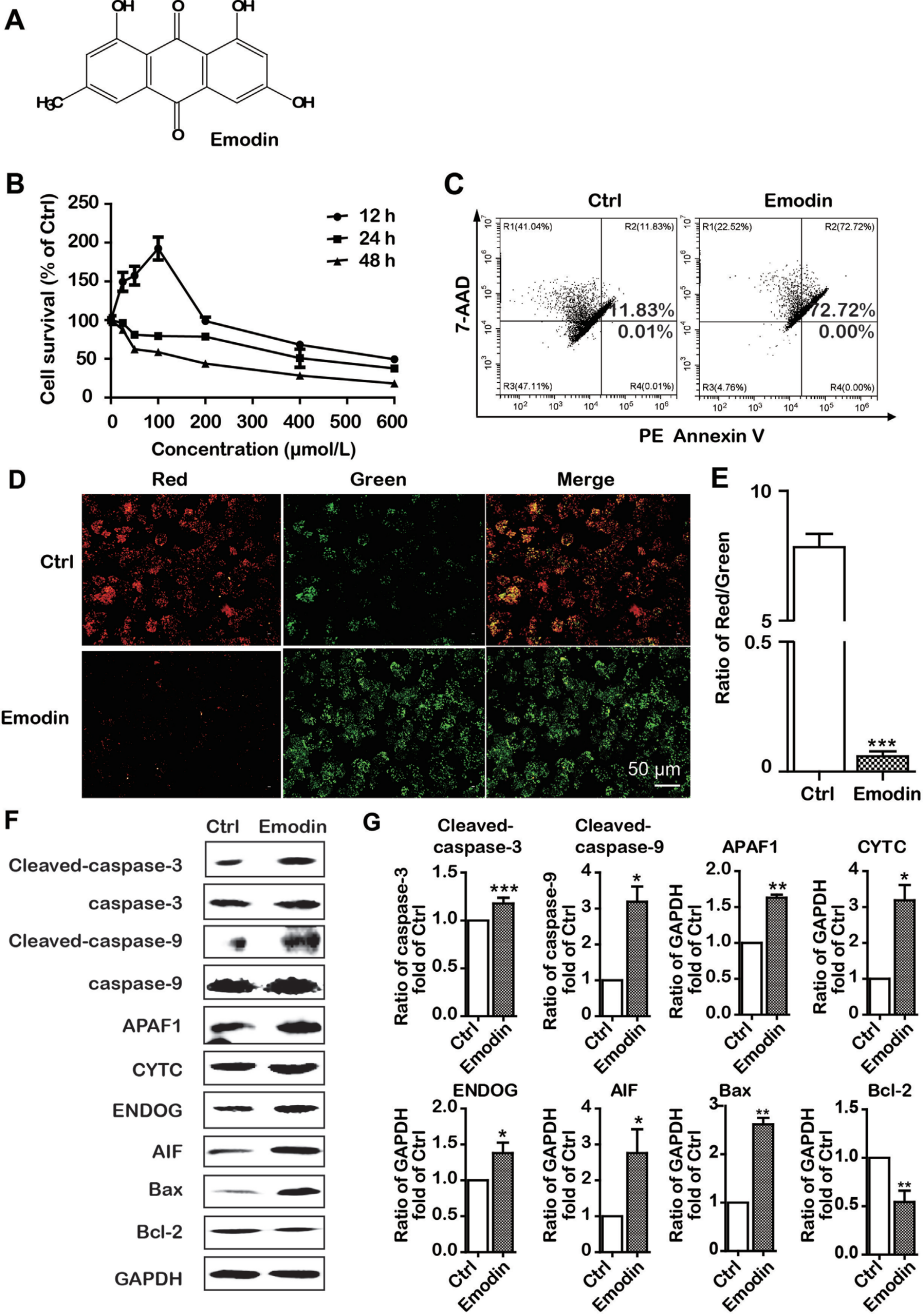
Emodin inhibited cell proliferation and induced intrinsic apoptosis in Bel-7402 cells. **(A)** The chemical structure of emodin. **(B)** Bel-7402 cell viability after treatment with emodin determined by the MTT assay. **(C)** Apoptosis in emodin-treated Bel-7402 cells as assessed by flow cytometry. **(D** and **E)** The fluorescence images with JC-10 staining in Bel-7402 cells. Bel-7402 cells were exposed to emodin (100 μmol/L) for 24 h followed by 30-min incubation with JC-10. When the mitochondrial membrane potential (ΔΨm) was higher, the JC-10 accumulated in the mitochondria matrix to form the polymer producing red fluorescence; otherwise, the green fluorescence from the JC-10 monomers was used to represent the cells that lost ΔΨm. Quantitative data of the ratio of red and green fluorescence intensity were measured. **(F** and **G)** Expression level of intrinsic apoptosis-associated proteins was measured after emodin treatment by Western blotting analysis. Data were expressed as mean ± standard error of the mean. *P < 0.05, **P < 0.01, and ***P < 0.001 *vs.* ctrl group (0 μmol/L), respectively.

Emodin is a natural anthraquinone derivative (C_15_H_10_O_5_). Its chemical structure is shown in **Figure 2A**. To determine the antiproliferative effect of emodin in HCC cells, the HCC cells Bel-7402 were incubated with emodin in different concentrations for 12, 24, and 48 h, and cell viabilities were then examined using the MTT assay. We found that emodin profoundly reduced cell viability in a dose-dependent and time-dependent manner in Bel-7402 cells (**Figure 2B**). Thus, we selected the concentration of 100 μmol/L and 24-h incubation for further experiments.

We further investigated the emodin-induced apoptosis using PE Annexin V-7AAD apoptosis detection kit. The results of flow cytometric analysis showed that 24 h treatment with emodin (100 μmol/L) significantly increased the apoptosis as compared to the control group (72.72% *vs,* 11.84% (**Figure 2C**). Previous studies have shown that apoptosis induced by anticancer drugs involved two major signal pathways: the mitochondria-mediated intrinsic apoptosis pathway and the death receptor-mediated extrinsic apoptosis pathway (Galluzzi et al., 2012). Thus, we speculated that emodin had induced apoptosis *via* the intrinsic pathway. For validation, we investigated the alterations of ΔΨm in emodin-treated Bel-7402 cells. We found that ΔΨm was diminished in Bel-7402 cells when exposed to emodin (**Figure 2D**). Furthermore, we found that emodin upregulated the expressions of Bax and downregulated the expression of Bcl-2 (**Figure 2F and G**). These results also approved the intrinsic pathway of emodin-induced apoptosis in Bel-7402 cells.

Because the intrinsic apoptotic pathway is divided into caspase-dependent and caspase-independent pathways (Galluzzi et al., 2012), we performed Western blotting to further clarify the apoptotic pathway induced by emodin and found an upregulation of caspase-independent-associated proteins (AIF and ENDOG). Interestingly, emodin also increased the expression of caspase-dependent-associated proteins (CYTC, APAF1, cleaved-caspase-9, and cleaved-caspase-3) (**Figure 2F and G**). Our results suggested that emodin induced both caspase-dependent and caspase-independent intrinsic apoptosis in Bel-7402 cells.

### Emodin Decreased Lipid Metabolism in Bel-7402 Cells

**Figure 3:**
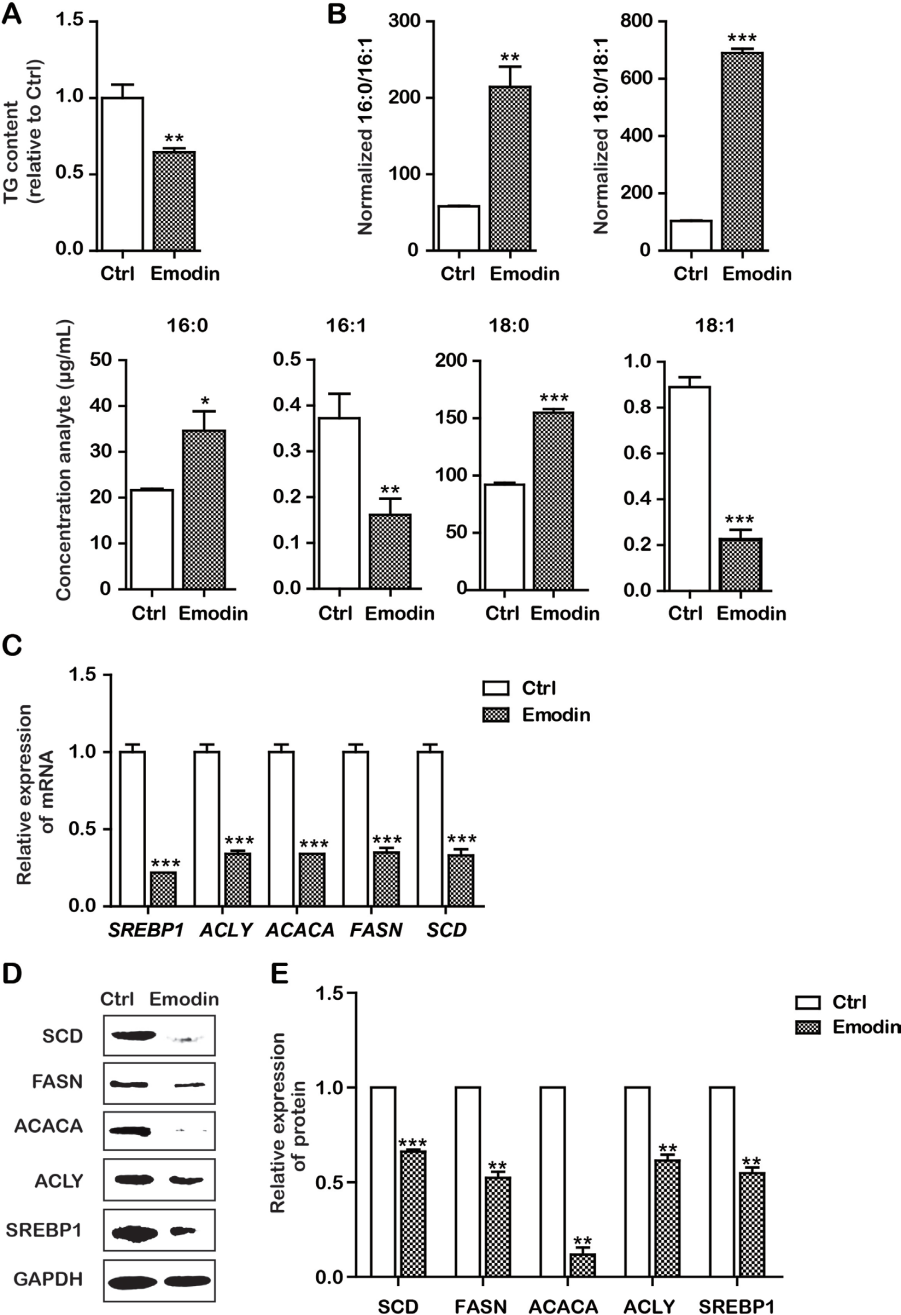
Emodin decreased lipid metabolism in Bel-7402 cells. **(A)** Total cellular TG content of Bel-7402 cells treated with emodin. **(B)** High-performance liquid chromatography-mass spectrometry (HPLC-MS) determination of the saturated fatty acids (SFAs) to monounsaturated fatty acids (MUFAs) ratios (palmitic acid to palmitoleic acid, 16:0 to 16:1) (stearic acid to oleic acid, 18:0 to 18:1) as well as the content of 16:0, 16:1, 18:0, and 18:1 in Bel-7402 cells treated with emodin. **(C)** The transcript level of lipid-associated genes in Bel-7402 cells when exposed to emodin. **(D** and **E)** The protein level of lipid-associated genes in Bel-7402 cells when exposed to emodin. Data were expressed as mean ± standard error of the mean. *P < 0.05, **P < 0.01, and ***P < 0.001 *vs.* ctrl group, respectively.

It has been reported that intrinsic apoptosis results from a bioenergetics and metabolic catastrophe coupled to multiple active executioner mechanisms. We then attempted to observe the effects of emodin on fatty acid metabolism. To determine the effect of emodin on *de novo *lipogenesis in Bel-7402 cells, we first examined the level of TG in Bel-7402 cells. Our data indicated that emodin decreased production of TG (**Figure 3A**). However, fatty acid metabolism includes the synthesis and desaturation of fatty acid. We presumed that emodin could alter the cellular lipid composition by restricting lipid desaturation. We therefore assessed the effect of emodin on cellular lipid composition by UPLC-MS. It should be mentioned that the UPLC-MS method employed here does not permit the definition of positional isomers. By doing so, we observed marked augments in the percentage of SFAs within MUFAs in emodin-treated cells. Likewise, we also found that the corresponding increase in palmitic acid and stearic acid and the reductions in palmitoleic acid and oleic acid (**Figure 3B**). These results emerged as alterations of cellular lipid composition by blocking lipid desaturation in emodin-treated Bel-7402 cells. To illuminate the molecular mechanisms by which emodin regulated the synthesis and desaturation of fatty acids, we assessed the expression of some lipid metabolism associated-genes involved in this process. Data showed that the mRNA expression of *SREBP1* was significantly downregulated in Bel-7402 cells after treatment with emodin (**Figure 3C**). To strengthen this evidence, we analyzed the expression of the key enzymes of fatty acid synthesis and desaturation that are regulated by SREBP1, including ACLY, ACACA, FASN, and SCD. Emodin dramatically attenuated the mRNA expression of *ACLY, ACACA, FASN,* and *SCD* in Bel-7402 cells (**Figure 3C**). These results were concordant with that of decreased protein levels of SREBP1, ACLY, ACACA FASN, and SCD in emodin-treated Bel-7402 cells (**Figure 3D**), indicating that emodin decreased lipid metabolism in Bel-7402 cells.

### SREBP1 Played a Pivotal Role in Maintaining Lipid Metabolism and Partially Avoiding Intrinsic Apoptosis in Bel-7402 Cells

**Figure 4:**
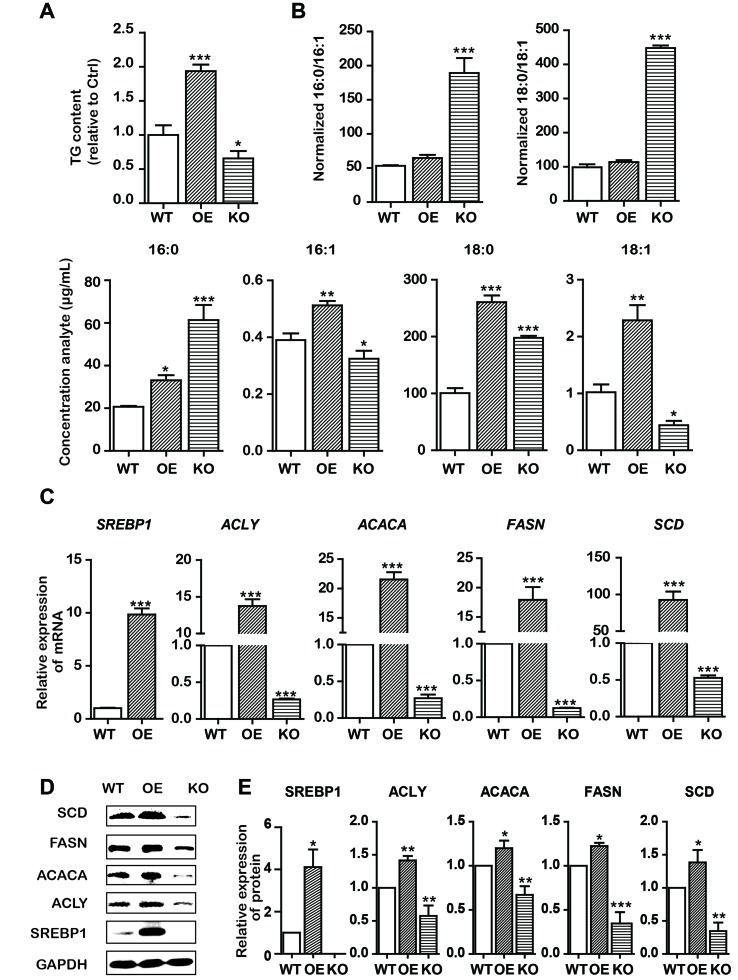
KO and OE of SREBP1 influenced lipid metabolism in Bel-7402 cells. **(A)** Total cellular TG content of SREBP1-KO, SREBP1-OE, and WT Bel-7402 cells. **(B)** HPLC-MS determination of the SFAs to MUFAs ratios (palmitic acid to palmitoleic acid, 16:0 to 16:1) (stearic acid to oleic acid, 18:0 to 18:1) as well as the content of 16:0, 16:1, 18:0, and 18:1 in SREBP1-KO, SREBP1-OE, and WT Bel-7402 cells. **(C)** The transcript level of lipid-associated genes in SREBP1-KO, SREBP1-OE, and WT Bel-7402 cells. **(D** and **E)** The protein level of lipid-associated genes in SREBP1-KO, SREBP1-OE, and WT Bel-7402 cells. Data were expressed as mean ± standard error of the mean. *P < 0.05, **P < 0.01, and ***P < 0.001 vs. WT group, respectively.

Based on the aforementioned results, we assumed that the effects of emodin on lowering lipid metabolism and inducing intrinsic apoptosis would involve interaction with SREBP1. To further support this assumption, we subsequently constructed stable OE/KO *SREBP1* Bel-7402 cell lines (**Figure 1**). Compared with the wild type (WT) control group, the synthesis of TG was decreased (**Figure 4A**), and the ratio of SFAs to MUFAs was decreased in SREBP1-KO (**Figure 4B**). Furthermore, the mRNA and protein expression of fatty acid synthesis genes (ACLY, ACACA, and FASN) and fatty acid desaturation gene SCD was downregulated (**Figure 4C and D**).

**Figure 5:**
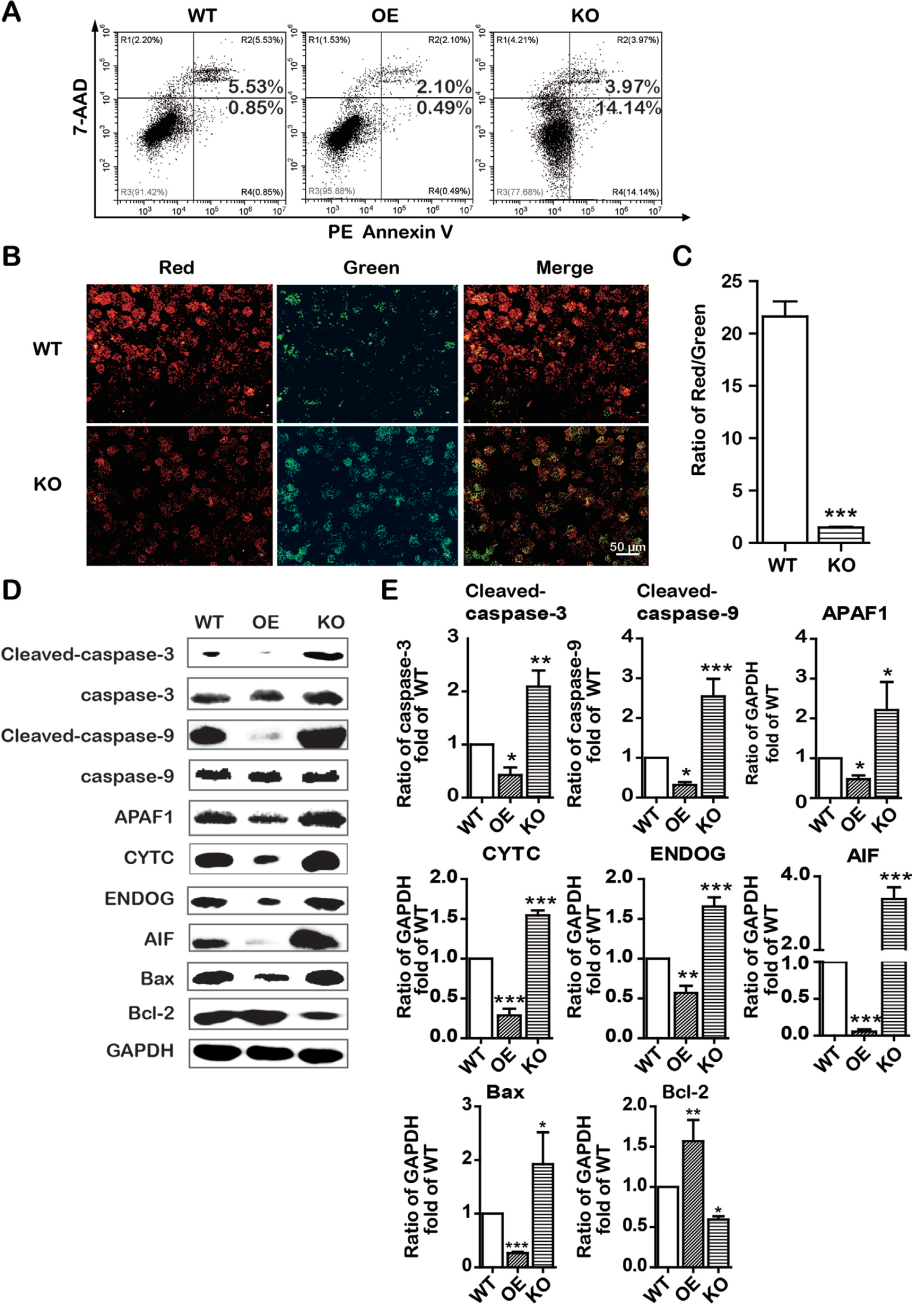
KO and OE of SREBP1 influenced intrinsic apoptosis in Bel-7402 cells. **(A)** Apoptotic cells were detected by the PE Annexin V-7AAD double staining assay in SREBP1-KO, SREBP1-OE, and WT Bel-7402 cells. **(B** and **C)** The ΔΨm was detected by JC-10 assay in SREBP1-KO, SREBP1-OE, and WT Bel-7402 cells. **(D** and **E)** The expression of intrinsic apoptosis associated-proteins in SREBP1-KO, SREBP1-OE, and WT Bel-7402 cells. Data were expressed as mean ± standard error of the mean. *P < 0.05, **P < 0.01, and ***P < 0.001 vs. WT group, respectively.

Similarly, to explore the role of SREBP1 in apoptosis induction, we examined the apoptosis in stable SREBP1-KO, SREBP1-OE, and WT Bel-7402 cell lines. Our data clearly indicated that KO of SREBP1 induced apoptosis (**Figure 5A**) and caused ΔΨm decline (**Figure 5B**) compared with that of WT Bel-7402 cells. Furthermore, compared with WT cells, the results of Western blot analysis indicated that the expression of intrinsic apoptosis-associated proteins (cleaved-caspase-3, cleaved-caspase-9, APAF1, CYTC, ENDOG, and AIF) was activated, and Bcl-2 was repressed in SREBP1-KO cell lines (**Figure 5D and E**). Interestingly, the apoptosis rate in SREBP1-KO cells (**Figure 5A**) was significantly less than that in emodin-treated cells (18.11% *vs*. 72.72%) (**Figure 2C** ****and **5A**), indicating SREBP1 was not the unique signaling pathway for cell survival and emodin may also induce cell apoptosis through other SREBP1-independent pathways.

### Emodin Decreased Lipid Metabolism and Induced Apoptosis by SREBP1-Dependent and -Independent Ways

**Figure 6:**
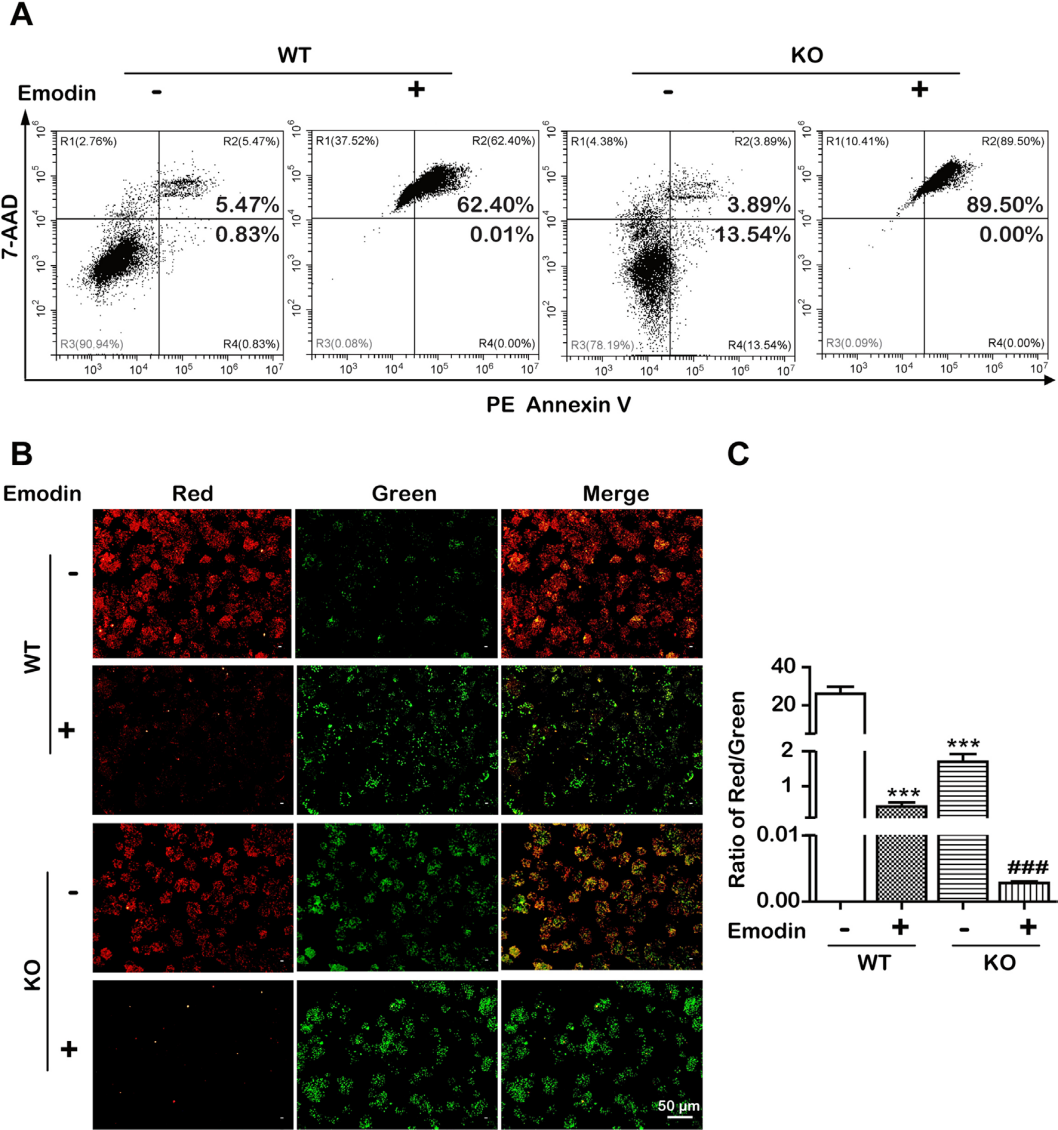
Emodin induced SREBP1-dependent and -independent apoptosis. **(A)** Apoptotic cells were detected using the PE Annexin V-7AAD double staining assay in SREBP1-KO and WT Bel-7402 cells after treatment with emodin. **(B** and **C)** The ΔΨm was detected using JC-10 assay in SREBP1-KO and WT Bel-7402 cells. Data were expressed as mean ± standard error of the mean. ***P < 0.001 *vs.* WT group, respectively. ^###^ *P* < 0.001 *vs.* SREBP1-KO group, respectively.

**Figure 7:**
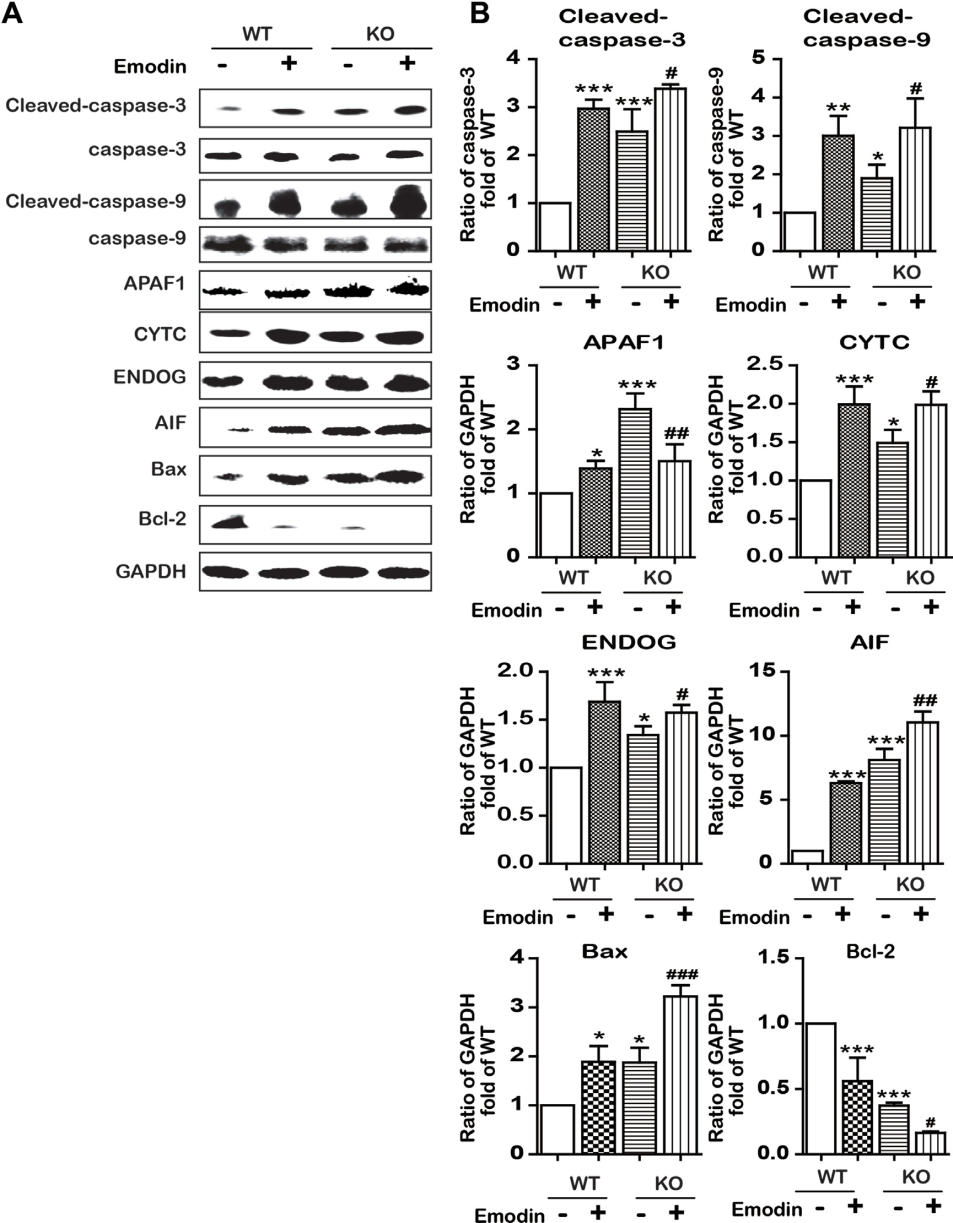
Emodin activated apoptosis-associated proteins by blocking in a SREBP1-dependent and SREBP1-independent manner. **(A** and **B)** The expression of intrinsic apoptosis-associated proteins in SREBP1-KO and WT Bel-7402 cells. Data were expressed as mean ± standard error of the mean. *P < 0.05, **P < 0.01, and ***P < 0.001 *vs.* WT group, respectively. ^#^ *P* < 0.05, ^##^ *P* < 0.01, and ^###^ *P* < 0.001 vs. KO group, respectively.

To examine whether emodin induced apoptosis in an SBREP1-dependent manner, we investigated apoptosis induction in emodin-treated *SREBP1*-KO cell lines. As shown in **Figure 6**, compared with SREBP1-KO cells, emodin combined with SREBP1-KO induced more apoptosis and decreased more mitochondrial membrane potential. In addition, the activation of caspase-dependent and caspase-independent intrinsic apoptosis-associated proteins was enhanced in emodin-treated SREBP1-KO cell lines (**Figure 7**). These results indicated that emodin induced apoptosis through both SREBP1-dependent and SREBP1-independent pathways in Bel-7402 cells.

## Discussion and Conclusion

According to statistical data from 2018, liver cancer is universally diagnosed and identified as a leading cause of cancer death, it is ranked fourth among common malignant tumors in global cancer mortality in which HCC accounted for 70% to 90% of mortality cases (Torre et al., 2015; Bray et al., 2018). The dysregulation of energy metabolism and resistance to cell death have recently been recognized as one of the most important hallmarks of HCC (Hanahan and Weinberg, 2011). The coordinated synthesis of macromolecules, including proteins and lipids, are the requirements for cell growth and survival (Griffiths et al., 2013). As compared with cells in most other normal tissues in humans, solid tumors, including HCC cells, can be exposed to low concentrations of nutrients and oxygen due to the ineffective vascular network (Lewis et al., 2015). Interestingly, the normal cells generally rely on the uptake of lipids from the circulation, whereas tumors often obtain the ability to make their own lipids (Menendez and Lupu, 2007). In additions, alteration in cellular metabolism constantly noted in cancer is the enhanced capacity of *de novo* lipid synthesis (Ricoult et al., 2016). Recent studies have drawn significant attention to metabolic reprogramming, especially lipid metabolism alteration, which is regarded as the initiating factor of tumor pathogenesis and progression. Continuous *de novo* lipogenesis is frequently activated in cancers, thereby providing extra lipids and lipid precursors during rapid cell proliferation (Martinez-Outschoorn et al., 2017). In the vital role of lipid metabolic reprogramming in cancer cell biology, the molecular programs and pathways that support the cancer metabolic process remain elusive; therefore, it is crucial to identify molecular mechanisms of lipid metabolism in tumorigenesis and tumor progression in HCC cells.

Because of the high recurrence of HCC after surgery and resistance to chemotherapy, traditional Chinese medicines used for cancer treatment have gained increasing attention, including He Shou Wu (Lin et al., 2015). As the main component of He Shou Wu, emodin induces both extrinsic and intrinsic apoptosis (Cui et al., 2016). However, it remains unclear whether a decrease in lipids is associated with cancer treatment. In this study, we found that emodin has significant antitumor activity as it suppressed cell viability and triggered apoptosis in Bel-7402 cells (**Figure 2B and C**). Based on the latest definition of cell death issued by the Nomenclature Committee on Cell Death (NCCD), the apoptosis is divided into intrinsic apoptosis and extrinsic apoptosis. The intrinsic apoptosis is mainly relevant to a mitochondrion-centered control mechanism (Galluzzi et al., 2012). After the ΔΨm dissipates, the apoptosis will be nonreversible. Our data showed that emodin distinctly decreased mitochondrial membrane potential, indicating a functional injury of mitochondria (**Figures 2D and E**). Furthermore, our data showed that the ratio of the protein expression of Bax/Bcl-2 was decreased, and caspase-9, APAF1, and CYTC were increased in emodin-treated Bel-7402 cells, this suggested that emodin induced intrinsic apoptosis. In addition, the intrinsic apoptosis has two pathways: one is caspase-dependent through releasing CYTC along with APAF1, triggering the caspase-9 to caspase-3 proteolytic cascade. The other is a caspase-independent pathway with AIF and ENDOG functions (Galluzzi et al., 2012). In this study, emodin also activated AIF and ENDOG, indicating that emodin induced apoptosis in both caspase-dependent and caspase-independent pathways.

Subsequently, we explored the underlying mechanisms of induction of intrinsic apoptosis by emodin. Here, we found that emodin acted as a potent inhibitor of SREBP1 and the downstream genes including *ACLY*, *ACACA*, and *FASN*. We observed substantial reduction in ACLY, ACACA, FASN, and SCD mRNA and protein expression in emodin-treated Bel-7402 cells, finally leading to the decrease in fatty acid biosynthesis (**Figure 3**). Furthermore, KO SREBP1 induced apoptosis and OE SREBP1 reversed this apoptotic process (**Figures 4** and **5**). These results strongly suggested that emodin induced intrinsic apoptosis in a SREBP1-dependent manner. In addition, interestingly, we observed that emodin alone and, combined with SREBP1-KO, induced more apoptosis than SREBP1-KO alone (**Figures 2C**, **5A**, **6A**, and **7**). Based on the evidence that emodin induced apoptosis in previous studies (Wang et al., 2013; Yu et al., 2013; Cui et al., 2016), this suggested that emodin may also induce apoptosis through a SREBP1-independent pathway in Bel-7402 cells. With regard to the effects of emodin on lipid metabolism in other types of cancer cells, Lee et al. reported that emodin inhibited proliferation and induced apoptosis by blocking FASN at a concentration of 10 to 50 μmol/L in colon cancer HCT116 cells (Lee et al., 2017), and Dong found that emodin could reduce FASN in a dose-dependent manner (20, 40, and 80 μmol/L) in liver cancer HCCLM3 cells (Dong, 2007). Li et al. reported chrysophanol, an analogue of emodin, inhibited liver cancer Huh-7 cells by suppressing expression of SREBPs and the downstream genes, FASN, ACACA, ACLY, SCD, and HMGCR at a concentration of 40 μmol/L (Li et al., 2015). In this study, the IC_50_ value for emodin on HCC Bel-7402 cells was around 80 μmol/L, indicating that different types of cancer cells require different inhibitory concentrations. Our data were consistent with the previous studies on emodin against other types of cancer cells.

Regarding the toxicity of emodin on normal human liver cells, Cui found that emodin at a concentration of 80 μmol/L could inhibit liver cancer HepG2 cells with a cell viability of 45.07% in 24 h; however, 100 μmol/L of emodin had no toxicity on human normal HL-7702 cells (Cui, 2016). Zhang reported 6.25 and 12.5 μg/L (about 23.15 and 46.30 μmol/L, respectively) of emodin showed no toxicity on L02 cells in 24 and 48 h, whereas 25 and 50 μg/L (about 92.59 and 185.18 μmol/L, respectively) of emodin induced S phase arrest and apoptosis in HL-7702 cells (Zhang et al., 2010). Our previous study also found that IC_50_ value for emodin on L02 cells was 36.69 μg/L (about 135 μmol/L) (Li et al., 2017). Compared with the aforementioned IC_50_ value (10 to 80 μmol/L) for emodin on cancer cells, the toxicity of emodin on human normal cells (with a IC_50_ value from 92.59 to 185.18 μmol/L) was slightly less.

In summary, our study demonstrated that emodin inhibited SREBP1-dependent and SREBP1-independent cell proliferation and resulted in caspase-dependent and caspase-independent intrinsic apoptosis induction in HCCs. Emodin might have a high potential for targeting SREBP1 in HCC.

## Author Contributions

XW and FC designed the study; NY, HL, and ML conducted the experiments; NY, CL, XW, and XC analyzed the data; NY, CL, XW, and ML wrote the manuscript; NY, YF, XW and ML revised manuscript. All authors read and approved the final manuscript.

## Funding

The study was financially supported by the National Natural Science Foundation of China (81874356, 31701294); the Young Scientist Innovation Team Project of Hubei Colleges (T201510); the Hubei Province Health and Family Planning Scientific Research Project (WJ2017Z023); the Open Project of Hubei Key Laboratory of Wudang Local Chinese Medicine Research, Hubei University of Medicine (WDCM2018002); the Key Discipline Project of Hubei University of Medicine and the Foundation for Innovative Research Team of Hubei University of Medicine (2014CXG03, 2018YHKT01); the Key Discipline Project of Hubei Province (2014XKJSXJ18); and the Start-up Foundation of Hubei University of Medicine (2017QDJZR26).

## Conflict of Interest Statement

The authors declare that the research was conducted in the absence of any commercial or financial relationships that could be construed as a potential conflict of interest.
